# Integration of Bulk RNA Sequencing and Single-Cell RNA Sequencing to Reveal Uveal Melanoma Tumor Heterogeneity and Cells Related to Survival

**DOI:** 10.3389/fimmu.2022.898925

**Published:** 2022-07-05

**Authors:** Guohong Gao, Aijun Deng, Shan Liang, Shengsheng Liu, Xinyi Fu, Xiaoyan Zhao, Zhilong Yu

**Affiliations:** Department of Ophthalmology, Affiliated Hospital of Weifang Medical University, Clinical Medical Institute, Weifang Medical University, Weifang, China

**Keywords:** uveal melanoma, single-cell RNA sequencing, RNA sequencing, tumor heterogeneity, transcription factors

## Abstract

Molecular classification based on transcriptional characteristics is often used to study tumor heterogeneity. Human cancer has different cell populations with distinct transcription in tumors, and their heterogeneity is the focus of tumor therapy. Our purpose was to explore the tumor heterogeneity of uveal melanoma (UM) through RNA sequencing (RNA-seq) and single-cell RNA sequencing (scRNA-seq). Based on the consensus clustering assays of the prognosis-related immune gene set, the immune subtype (IS) of UM and its corresponding immune characteristics were comprehensively analyzed. The heterogeneous cell groups and corresponding marker genes of UM were identified from GSE138433 using scRNA-seq analysis. Pseudotime trajectory analysis and SCENIC analysis were conducted to explore the trajectory of cell differentiation and the regulatory network of single-cell transcription factors (TFs). Based on 37 immune gene sets, UM was divided into two different immune subtypes (IS1 and IS2). The two kinds of ISs have different characteristics in prognosis, immune-related molecules, immune score, and immune cell infiltration. According to 11,988 cells of scRNA-seq data from six UM samples, 11 cell clusters and 10 cell types were identified. The subsets of C1, C4, C5, C8, and C9 were related to the prognosis of UM, and different TF–target gene regulatory networks were involved. These five cell subsets differentiated into 3 different states. Our results provided valuable information about the heterogeneity of UM tumors and the expression patterns of TFs in different cell types.

## Introduction

Melanoma derives from the genetic mutations in melanocytes, existing in the skin, eye, inner ear, and leptomeninges (1–3). Uveal melanoma (UM) is a rare ocular malignant tumor with a rapidly increasing incidence worldwide (4). The current eye-sparing treatment options include surgical treatment, plaque brachytherapy, proton beam radiotherapy, stereotactic photon radiotherapy, or photodynamic therapy (5). Even with successful local treatment of primary tumors, more than 50% of UM patients will develop metastatic diseases (6, 7). For patients, there is no effective treatment option so far. The median overall survival (OS) time is 10–13 months, and the cure rate is close to zero (8). Improving the survival rate of metastatic UM is the main clinical goal at present. For this purpose, a number of studies have developed a series of prognostic biomarkers for UM. For example, Wang et al. discovered MMP1 and MMP9 as potential biomarkers for predicting UM OS and disease-free survival (9).

Although prognostic biomarkers help predict UM prognosis, understanding the molecular mechanism of UM development is also a central work for developing potential drug targets for UM treatment. Intratumor heterogeneity is considered to be one of the main determinants of metastasis, drug resistance, and recurrence (10). Human tumor possesses a complex ecosystem, comprising malignant/transformed cells and a plethora of different cell types recruited from the surrounding tissue and immune system (11). Different heredity, epigenetics, transcriptome, proteome, and functional characteristics of different cells in this system are important factors that make up tumor heterogeneity (12). The fine characterization of these tumor heterogeneity levels is greatly important in a successful treatment of cancer. At present, the heterogeneity of UM metastasis has not been fully studied.

Single-cell RNA sequencing (scRNA-seq) is a useful tool for analyzing the characteristics of various cell types in and around tumors, and it provides high-throughput and high-resolution transcriptome analysis of individual cells, which can facilitate a deeper understanding of the diversity of cell states and heterogeneity of cell populations (13). Moreover, the scope of application of scRNA-seq includes the identification of stem cells, the discovery of new biomarkers, and the detection of transcriptome tracks in cell populations in response to drug therapy over time (14). Lines of studies have revealed disease evolution, heterogeneity, and prognostic genes of UM based on single-cell analysis (10). Durante et al. discovered that one of the immune checkpoints, LAG3, was a potential candidate for immune checkpoint blockade in metastatic UM patients (15). The transcription factor (TF), HES6, was identified as a key driver for metastatic UM from scRNA-seq data (16). Single-cell data can also reveal the intra- and intertumoral heterogeneity of UM in different metastases of a patient, promoting the understanding of metastatic complexity (17). However, the molecular mechanism of UM development remains to be clarified.

In this study, we explored the intratumor heterogeneity of UM through the combination of scRNA-seq and bulk RNA-seq. We constructed two molecular subtypes or immune subtypes with distinct prognosis based on immune-related gene sets and bulk transcriptome data of UM. Eleven cell clusters were identified from scRNA-seq data, with five clusters being significantly associated with UM prognosis. We established gene–TF and mRNA–lncRNA interaction networks, and screened six hub genes and three key lncRNAs that may potentially contribute to UM development. Briefly, our work provided a new direction for understanding the regulatory mechanism of UM through interpreting the heterogeneity from scRNA-seq data.

## Materials and Methods

### Downloading and Processing of Raw Data

The workflow of this study is shown in **Figure S1**. UM samples containing expression data with fragments per kilobase million (FPKM) format were downloaded from The Cancer Genome Atlas (TCGA, http://tcga-data.nci.nih.gov/tcga/) database (named as TCGA cohort), and FPKM was converted into transcripts per kilobase (TPM) format. UM samples with clinical information and gene expression data of GSE22138 cohort were downloaded from the Gene Expression Omnibus (GEO, https://www.ncbi.nlm.nih.gov/) database of NCBI. UM samples without survival time or survival status were excluded in both two cohorts. After preprocessing, 80 and 63 UM samples remained in TCGA and GSE22138 cohorts, respectively (**Tables S1, S2**). Limma R package (18, 19) was used to normalize the quantile of gene expression data.

### UM Classification Based on Immune Score

The enrichment score of 100 immune gene sets was calculated for each sample in the TCGA cohort by the calculate_sig_score function in the IOBR package (20). Univariate Cox regression analysis was performed to screen immune genes sets associated with OS using the coxph function of the survival package (https://mran.microsoft.com/web/packages/survival/index.html) under the criteria of *p* < 0.001. Then, immune gene sets with *p* < 0.001 served as a basis for conducting consensus clustering through the ConsensusClusterPlus R package (21). The clustering process with 500 bootstraps was performed by sampling 90% of the data in each iteration. The number of clusters was set to 2–10, and the optimal number of clusters was determined by the cumulative distribution function (CDF) curves and area under the CDF curve. The obtained clusters were compared with OS by Kaplan–Meier survival analysis.

### Tumor Microenvironment Characteristics of Immune Subtypes

The tumor microenvironment (TME) among immune subtypes (ISs) was compared from different aspects. The expression of immune-related molecules, including chemokines, chemokine receptors and 47 immune checkpoints, was calculated for each IS (22), and the differential expression among ISs was analyzed. TME-related signatures including IFN-γ score (22), CYT score (23), and angiogenesis score (24) were obtained from previous research, and their enrichment scores were calculated through single-sample gene set enrichment analysis (ssGSEA) (25). ESTIMATE algorithm (26) was performed to estimate immune infiltration and stromal infiltration. CIBERSORT (27) was conducted to evaluate the estimated proportion of 22 immune cells. The gene sets of ten oncogenic pathways including cell cycle, Hippo, Myc, Notch, NRF1, PI3K, TGF-β, Ras, TP53, and Wnt were obtained from a previous research (28).

### Marker Genes That Define Different ISs

The differential expression between the two ISs were analyzed by the limma package (18). The differentially expressed genes (DEGs) between the two ISs were screened under |log2 (Fold Change)| > 1 and adjusted *p*-value (*q*-value) < 0.05. The gene significantly upregulated in IS1 was defined as the characteristic gene of IS1, and the gene significantly upregulated in IS2 was used as the marker gene of IS2.

### Acquisition and Analysis of scRNA-Seq Data

The scRNA-seq data of six UM tumor samples were also downloaded from the GEO database (entry number GSE138433). Firstly, the “Seurat: package was used to process the scRNA-seq data (29). The percentage of mitochondrial genes was calculated by the PercentageFeatureSet function, and the quality control standard was set as follows: the number of genes expressed in each cell was more than 500 but less than 7,000, and the content of mitochondria was less than 35%. The data of the sample were merged and standardized by log-normalization. The top 2,000 highly variant genes were screened according to the FindVariableFeatures function. Scale and principal component analysis (PCA) were performed according to the RunPCA function based on high variation genes. Then, the cells were clustered by the FindNeighbors and FindClusters functions. The top 40 principal components were selected to further reduce the dimension using t-distributed stochastic neighborhood embedding (t-SNE). FindAllMarkers was used to identify marker genes in each cluster.

### Trajectory Analysis of Cell Clusters

Single-cell pseudotime trajectories of UM cells were constructed using “Monocle 2”, which learns complex cellular trajectories with multiple branches in a fully data-driven manner (30). It is generally believed that the cells on the same branch have the same differentiation state, while the cells located in different branches are considered to have different cell differentiation trajectories. BEAM algorithm was applied to mine key genes in the cell development within the trajectory. The top 100 key genes were screened by false discovery rate.

### Construction of the mRNA–lncRNA Interaction Network

Through analyzing the difference of cell clusters among IS, the significant cell clusters between different ISs were screened and their marker genes were extracted. The starBaseV3.036 database was designed to integrate large-scale CLIP-Seq (HITS-CLIP, PAR-CLIP, iCLIP, and CLASH) data to decode the interaction network (31). Specifically, 1,055,319 miRNA–mRNA pairs containing 484 miRNA and 15,064 mRNA and 63,698 miRNA–lncRNA interaction data of 642 miRNA and 3,789 lncRNA were downloaded from starBase V3.0. According to the miRNAs shared by mRNAs and lncRNAs, the possibility of mRNA–lncRNA interaction was calculated using a hypergeometric test, and the *p*-value was calculated according to the following formula:


$$pvalue=1-\sum_{k=0}^{r-1}\frac{\left(\begin{array}{l} t\\ k \end{array}\right)\left(\begin{array}{l} m-t\\ n-k \end{array}\right)}{\left(\begin{array}{l} m\\ n \end{array}\right)}$$

In the formula, *m* represents the total number of miRNAs in the starBase database, *t* represents the number of miRNAs that interacted with the mRNA, *n* represents the number of miRNAs that interacted with the lncRNA, and *r* represents the number of miRNAs shared between mRNA and lncRNA.

### Data Accessibility

The RNA-seq data of UM samples were obtained from the TCGA (https://portal.gdc.cancer.gov/) database and GEO database (accession number: GSE22138, http://www.ncbi.nlm.nih.gov/geo/). The scRNA-seq data were also obtained from the GEO database (accession number: GSE138433).

### Statistical Analysis

All statistical analysis was implemented in R software (v4.0). In the TCGA dataset, the sample number (*n*) of IS1 and IS2 was 38 and 42, respectively. In the GSE22138 dataset, *n* (IS1) = 28 and *n* (IS2) = 32. Log-rank test was conducted in Kaplan–Meier survival analysis and Cox regression analysis. Student’s *t* test was conducted between the two groups. Chi-square test was conducted to compare the distribution of clinical features in two subtypes. *p* < 0.05 was considered as significant.

## Results

### The Classification of UM Patients Based on the Immune Gene Set Related to Prognosis Was Associated With Different Clinicopathological Features

**Figure 1:**
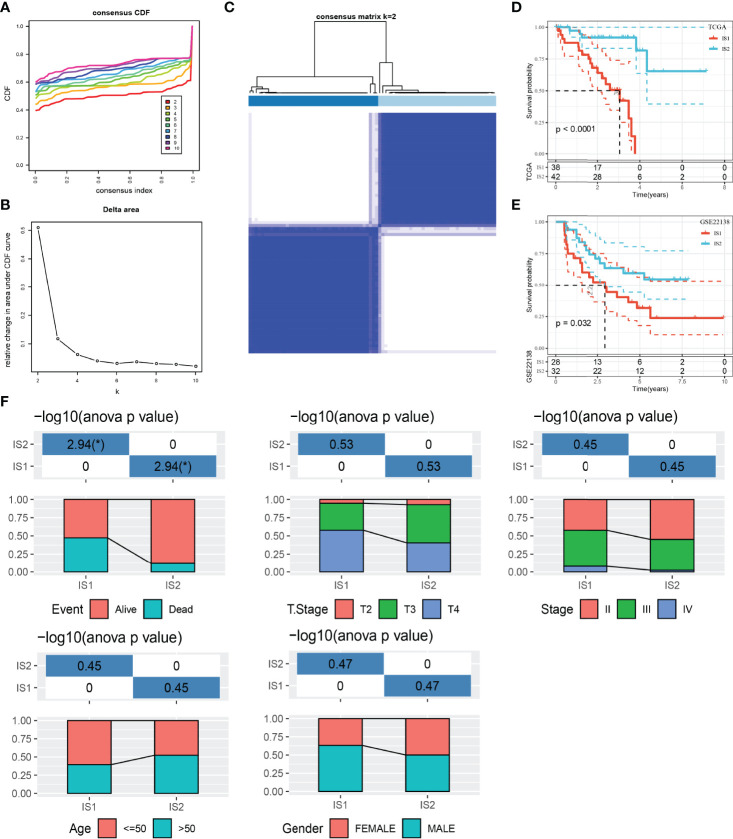
The relationship between classification of UM patients based on prognostic immune gene sets and different clinicopathological features. **(A)** CDF curves of the consensus score. **(B)** Relative change in area under the CDF curve. **(C)** Consensus clustering matrix for *k* = 2. **(D)** Survival analysis of IS1 (*n* = 38) and IS2 (*n* = 42) in the TCGA dataset. **(E)** Survival analysis of IS1 (*n* = 28) and IS2 (*n* = 32) in the GSE22138 dataset. **(F)** Clinicopathological characteristics of two ISs in the TCGA dataset, including age, gender, T stage, TNM stage, and survival status. *p < 0.05. CDF, cumulative distribution function.

We used IOBR package to quantify the enrichment scores of 100 immune gene sets for each sample in the TCGA dataset. Univariate Cox regression analysis was carried out to screen the gene sets related to prognosis from 100 immune gene sets (*p* < 0.001), and 37 immune gene sets related to UM survival were obtained. ConsensusClusterPlus was used to cluster 80 UM samples in TCGA. According to the optimal CDF curve and the delta area plot, *k* = 2, two molecular subtypes or ISs (IS1 and IS2) were produced (**Figures 1A–C**). Among the two UM subtypes of the TCGA dataset, the survival result of IS2 was significantly better than that of IS1 (**Figure 1D**). This result was also verified in the GSE22138 dataset (**Figure 1E**).

After that, the clinicopathological features of two ISs in the TCGA dataset were compared. The proportion of patients with age ≤ 50 years old and male patients in IS1 was more than 50%. IS2 patients were equally divided into men and women and age ≤50 and >50. Compared with IS2, the proportion of patients with T stage and TNM stage in the middle and late stage of IS1 was higher. The proportion of deaths in IS1 was also significantly higher than that in IS2 (**Figure 1F**).

### Correlation Between ISs and Immune-Related Indexes in TME

**Figure 2:**
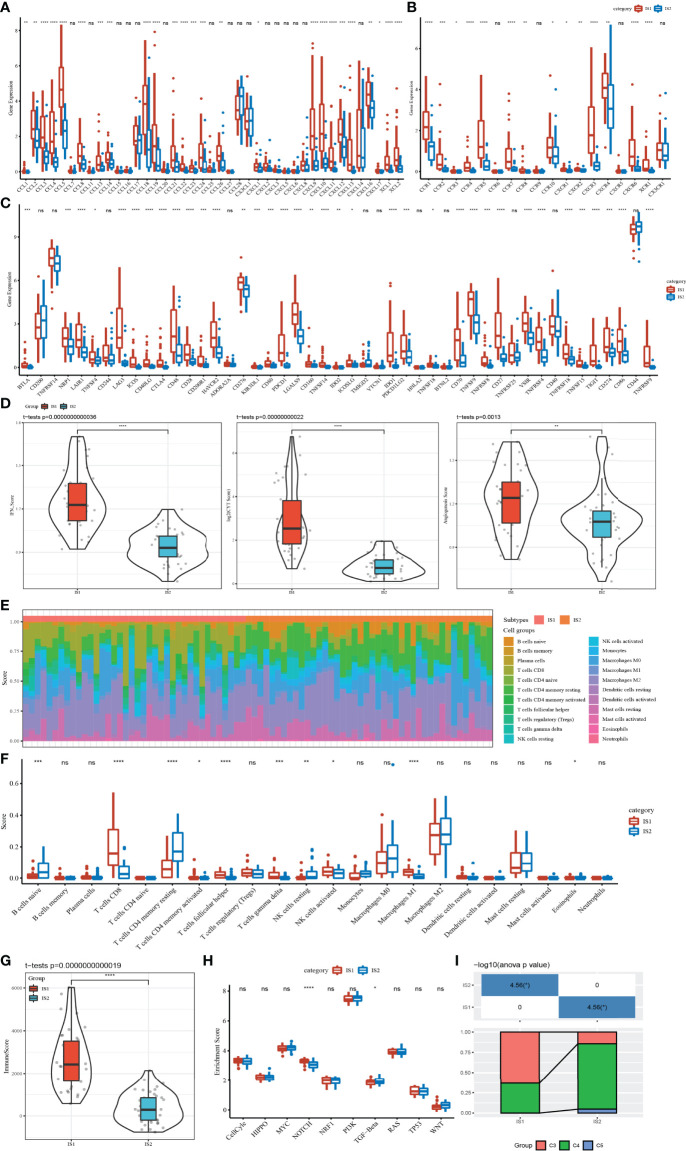
Correlation between ISs and immune-related indexes in the TCGA dataset. **(A)** Differential expression analysis of chemokines between IS1 and IS2. **(B)** Expression difference of chemokine receptors between two ISs. **(C)** The immune checkpoint of differential expression between IS1 and IS2. **(D)** IFNγ, CYT, and angiogenesis scores in IS1 and IS2. **(E)** The percentage score of infiltrating immune cells between the two ISs. **(F)** Differences in infiltrating immune cell scores between IS1 and IS2. **(G)** Enrichment scores of IS1 and IS2 on 10 signal pathways. **(H)** Immune score difference between IS1 and IS2. **(I)** Analysis of pan-cancer molecular subtypes included in IS1 and IS2. ns, no significance. *p < 0.05, **p < 0.01, ***p < 0.001, ****p < 0.0001.

TME, which is an important characteristic contributing to cancer development, is composed of a series of immune cells, stromal cells, and other inflammatory factors. To characterize the relationship between ISs and TME, the expression differences of chemokine, chemokine receptor, and immune checkpoint in two ISs were measured and compared at the molecular level. There were a large number of chemokine and chemokine receptors differentially expressed between IS1 and IS2. The expression level of almost all of them in IS1 was significantly higher than that in IS2 (**Figures 2A, B**). Among 47 immune checkpoints, we observed that 36 of them were differentially expressed between IS1 and IS2, with IS2 having a higher expression level of most immune checkpoints (**Figure 2C**). IS1 had significantly higher scores of IFN-γ, CYT, and angiogenesis compared to IS2 (**Figure 2D**). Two ISs exhibited different infiltration patterns of different immune cells (**Figure 2E**). In particular, CD8 T cells, helper follicular T cells, gamma delta T cells, and M1 macrophages were highly enriched in IS1 (*p* < 0.001), while resting memory CD4 T cells and resting NK cells were more significantly enriched in IS2 (*p* < 0.01, **Figure 2F**), with IS1 having an overall higher immune infiltration than IS2 (*p* < 0.0001, **Figure 2G**). Although IS1 had a higher CYT score and a higher enrichment of CD8 T cells, IS2 had worse prognosis, which may be resulting from its higher IFN-γ score, higher angiogenesis score, and immune checkpoint expression patterns. In addition, among 10 oncogenic pathways, only Notch and TGF-β signaling pathways were differentially activated between IS1 and IS2 (*p* < 0.05, **Figure 2H**). In particular, the enrichment score of the Notch signaling pathway was significantly higher in IS1 (*p* < 0.0001). By mapping the classic pan-cancer molecular subtypes (32) to our immune subtypes, we found that C3 and C4 subtypes consisted of the majority subtypes in IS1 and IS2 (*p* < 0.05, **Figure 2I**). In particular, the C3 subtype with a poor prognosis had a markedly higher proportion in IS1.

### Analysis of scRNA-Seq Data of UM Revealed 11 Cell Clusters

**Figure 3:**
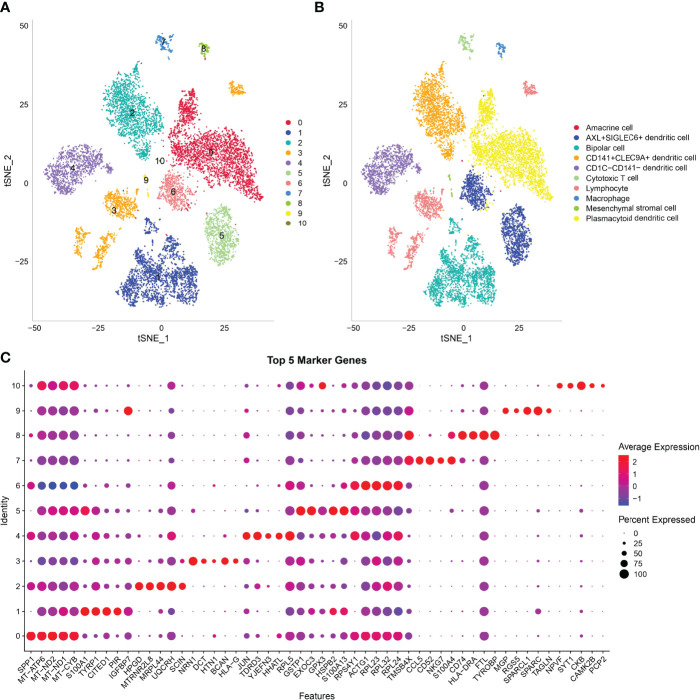
Analysis of scRNA-seq data in UM revealed 11 cell clusters annotated to different cell types. **(A)** The t-SNE map of all cells after quality control and standardization revealed 11 cell clusters marked with different colors. **(B)** 11 cell clusters according to gene marker annotated cell types. **(C)** The bubble diagram shows the expression of the top five marker genes in different cell clusters.

**Figure 4:**
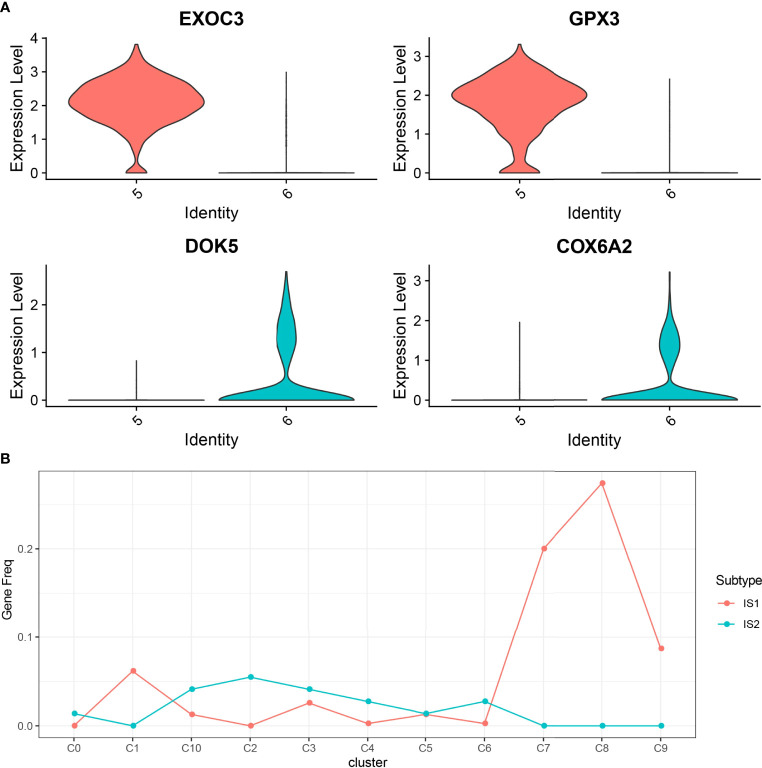
The relationship between 11 cell clusters and two ISs. **(A)** The violin picture shows the expression of C5 and C6 specific genes in two cell clusters. **(B)** The level of marker genes of 11 cell clusters in IS1 and IS2.

In this study, 17,850 scRNA-seq data from six UM samples were obtained from GSE138433. After quality control and standardization, 11,988 cells remained for analysis (**Figure S2**). The number of cells contained in each sample before and after quality control is shown in **Figure S3**. The analysis of genes in the cells showed high-frequency and low-frequency intercellular variation genes. The top 20 genes with the highest frequency variation among 2,000 low-frequency intercellular variation genes were labeled (**Figure S4**). PCA showed that there was no obvious batch effect of the six samples (**Figure S5**). All cells were visualized by t-SNE and clustered to specific cell types based on the expression of markers with known populations and every sample showed differences in cell composition (**Figure S6**). We identified 11 cell clusters from the whole single-cell map (**Figure 3A**). According to the expression pattern of the marker genes, C0 was annotated as plasmacytoid dendritic cell, C1 as bipolar cells, C2 as CD141+CLEC9A+ dendritic cells, C3 as lymphocytes, C4 as CD1C-CD141- dendritic cells, C5 and C6 as AXL+SIGLEC6+ dendritic cells, C7 as cytotoxic T cells, C8 as macrophages, C9 as mesenchymal stromal cells, and C10 as amacrine cells (**Figure 3B**). The top 5 marker genes of each cluster were shown in the bubble diagram, and each cell cluster had its own unique gene expression pattern different from other cell clusters (**Figure 3C**). For C5 and C6 annotated as AXL+SIGLEC6+ dendritic cells, the difference was that C5 specifically expressed EXOC3 and GPX3, and that C6 specifically expressed DOK5 and COX6A2 (**Figure 4A**).

To establish the relationship between ISs and cell clusters, the marker genes of IS1 and IS2 were defined. Differential expression analysis between IS1 and IS2 was performed by the limma package. We observed that 390 DEGs were significantly upregulated in IS1 and 73 DEGs were significantly upregulated in IS2 (**Figure S7**), and these upregulated genes were determined as their respective marker genes for the two subtypes. The expression of a total of 463 DEGs in 11 cell clusters was presented (**Figure S8**). We observed that these DEGs were obviously expressed in C1, C7, and C8. Subsequently, we analyzed the distribution of marker genes of 11 cell clusters in the markers of IS1 and IS2 to compare the similarity between clusters and subtypes (a higher proportion of intersected genes between the markers of clusters and subtypes indicates a higher similarity). C0, C2, C3, C4, C6, and C10 were more similar to IS2, and C1, C7, C8, and C9 were more similar to IS1 (**Figure 4B**).

### The Abundance of 11 Kinds of Cell Clusters in Different ISs and Their Effects on the Prognosis of UM

**Figure 5:**
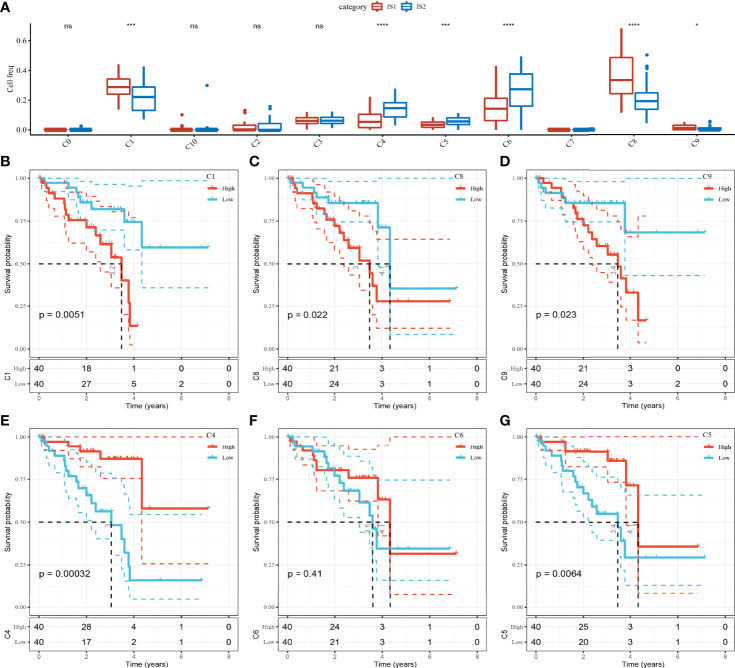
The abundance of 11 kinds of cell clusters in different ISs and their effects on the prognosis of UM. **(A)** The 11 cell subpopulations score differences between the two ISs. **(B–G)** Kaplan–Meier survival plots of UM patients grouped by C1, C4, C5, C6, C8, and C9 clusters. ns, no significance. *p < 0.05, ***p < 0.001, ****p < 0.0001.

To compare the abundance of 11 cell clusters in IS1 and IS2, we calculated the enrichment score of their marker genes in ISs by CIBERSORT. The results showed that there were significant differences in C1 (bipolar cells), C4 (CD1C-CD141- dendritic cells), C5 (AXL+SIGLEC6+ dendritic cells_1), C6 (AXL+SIGLEC6+ dendritic cells_2), C8 (macrophages), and C9 (mesenchymal stromal cells) subpopulation scores between IS1 and IS2 (**Figure 5A**). The UM samples were grouped according to the scores of six clusters, and the survival analysis between groups was carried out. The results showed that except for C6 clusters, the OS of patients in different groups could be significantly distinguished according to the scores of the other five clusters (C1, C4, C5, C8, and C9). Among the risk groups divided based on C1 or C8 or C9 cluster scores, the OS of patients with low scores was significantly longer than that of patients with high scores. In contrast, patients with high C4 cluster scores or high C5 cluster scores had significant survival advantages than patients with low C1 or C5 cluster scores (**Figures 5B–G**).

### Identification of 3 Subsets by Cell Trajectory Analysis of Five Cell Clusters

**Figure 6:**
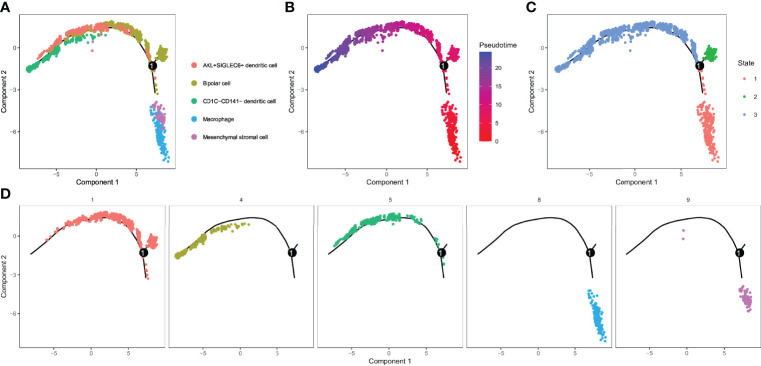
Identification of 3 subsets by cell trajectory analysis of five cell clusters. **(A–D)** Cell trajectory and pseudotime analysis for the five cell clusters (C1, C4, C5, C8, and C9) significantly correlated with overall survival of UM.

For the five cell clusters significantly related to UM prognosis, we performed pseudo-sequential analysis by monocle based on the expression of their marker genes. In the results, we noticed that five cell clusters (C1, C4, C5, C8, and C9) were projected onto three different states (**Figures 6A–D**). Bipolar cells (C1) distributed in all three states, indicating a high degree of its heterogeneity. CD1C-CD141- dendritic cells (C4) and AXL+SIGLEC6+ dendritic cells (C5) accumulated in state 3, while macrophages (C8) and mesenchymal stromal cells (C9) almost distributed in state 2. C8 and C9 were similar to IS1 according to the previous results, and they were located in the initial state, suggesting that the development of C8 and C9 may be related to poor prognosis. Then, we investigated the gene expression pattern variation according to the pseudotime for different cell clusters and states. The top 100 key genes were screened by the BEAM algorithm. Three states manifested different expression patterns as C1 was highly expressed in state 1 and state 2, C4 and C5 were highly expressed in state 3, and C8 and C9 were mostly expressed in state 2 (**Figure S9**).

### Identification of Transcription Factor–Target Gene Networks

**Figure 7:**
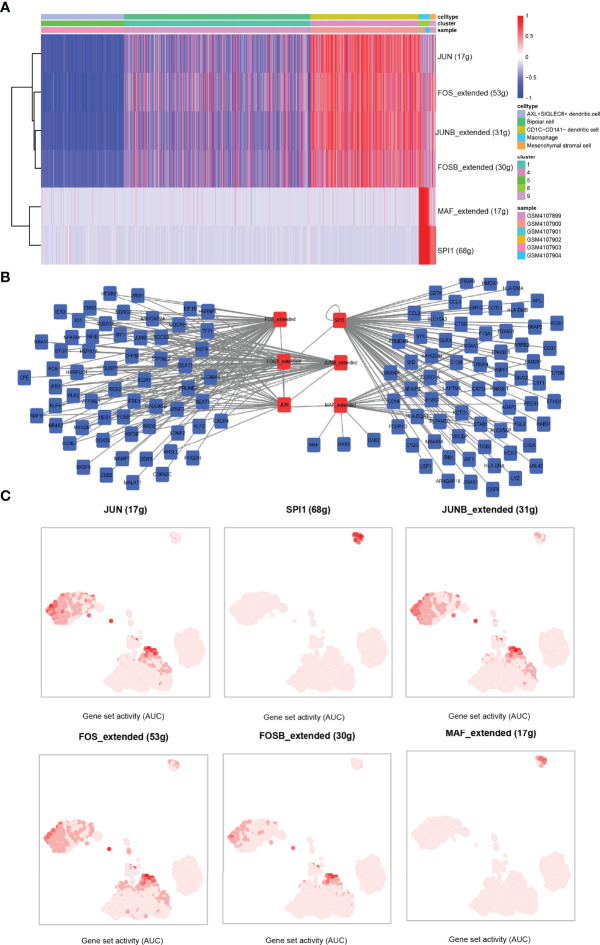
Identification of transcription factor–target gene interactions using SCENIC. **(A)** The heat map showed the enrichment of TFs in the five cell clusters, with blue representing AUC < 0 of *z*-score and red representing AUC > 0 of *z*-score. AUC values were normalized (*z*-score) according to cells (horizontal axis). **(B)** TF–target gene interaction network. **(C)** In the tSNE dimension, the darker the AUC of transcription factors in each cell cluster, the higher the AUC value. AUC, area under the ROC curve.

To study the activity of TFs in five cell subsets, single-cell transcriptional factor regulatory networks were clustered by SCENIC. A TF enrichment heat map showed that JUN was significantly enriched in CD1C-CD141- dendritic cells, and in this cell cluster, FOS, JUNB, and FOSB showed stronger activities. MAF and SPI1 were highly enriched in macrophage. FOS and JUNB and SPI1 were significantly enriched in mesenchymal stromal cells (**Figure 7A**). We constructed the TF–target gene network of these TFs, and noticed that JUNB, FOS, FOSB, and JUN shared many target genes, and that SPI1 and MAF had great common target genes (**Figure 7B**). Combined with the results of t-SNE, we suspected that JUN was a regulatory TF, and FOS specifically expressed in CD1C-CD141- dendritic cells was mainly expressed in CD1C-CD141-dendritic cells, macrophages, and mesenchymal stromal cells. SPI1 was the main regulatory TF of mesenchymal stromal cells. FOSB and JUNB were also important TFs in CD1C-CD141- dendritic cells. MAF was mainly expressed in macrophages and is a specific TF in macrophages (**Figure 7C**).

### Construction of the mRNA–lncRNA Interaction Network

**Figure 8:**
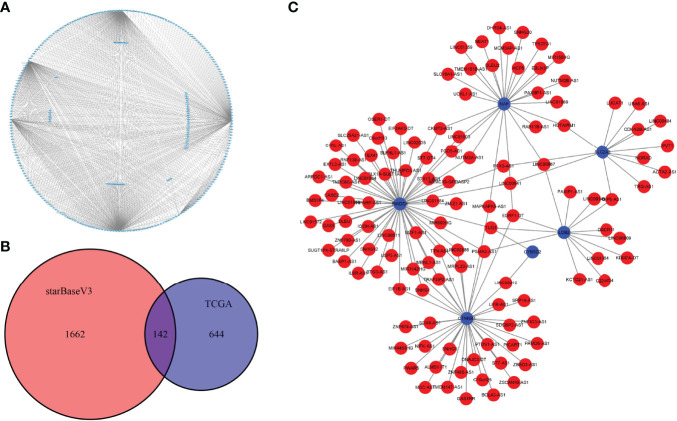
Construction of the mRNA–lncRNA interaction network. **(A)** The ceRNA global network of mRNA–lncRNA pairs with correlation coefficients >0.4 and *p* < 0.05 was analyzed by the Hmisc package. **(B)** The Venn diagram shows the intersection of the mRNA–lncRNA pairs obtained from the starBase V3.036 database and the mRNA–lncRNA pairs calculated by the Hmisc package. **(C)** The interaction network of 142 mRNA–lncRNA pairs; blue for mRNA and red for lncRNA.

LncRNAs are a group of regulatory RNAs and are commonly dysregulated in cancer. Therefore, we used the marker genes to predict potential lncRNAs involved in tumorigenesis to further understand the regulatory role of key cell clusters in the UM development. Based on the miRNA–mRNA pair and miRNA–lncRNA interaction data downloaded from starBase V3.036 database, we used the hypergeometric test to establish mRNA–lncRNA connection. In the expression profile of TCGA samples, the correlation of mRNA–lncRNA pairs was analyzed by the Hmisc packet, and the mRNA–lncRNA pairs with correlation coefficient > 0.4 and *p* < 0.05 were used to develop the ceRNA global network (**Figure 8A**). By taking the intersection of the mRNA–lncRNA pairs obtained from the starBase V3.036 database and the mRNA–lncRNA pairs calculated by the Hmisc package, 142 common mRNA–lncRNA pairs were obtained (**Figure 8B**). **Figure 8C** shows the interaction network of 142 mRNA–lncRNA pairs. It can be seen that six genes (MAF, RWDD1, SLC2A3, LDB2, CYB2D2, and CTNNB1) were the key regulators of mRNA–lncRNA interaction in UM.

## Discussion

Significant intratumor heterogeneity exists in both genomes and transcriptome of UM patients (33). The heterogeneity in one group may have a complex relationship with the heterogeneity in another group. The joint analysis of multiple groups in the same cell enables us to accurately clarify the characteristics and relationships between these layers in the tumor (34). In this study, we combined RNA-seq and scRNA-seq data to explore the immunophenotype and different cell subsets of UM and the relationship between them.

According to RNA-seq data, UM was divided into two types of ISs. The OS of IS1 was shorter than that of IS2, and at the molecular level, the expression levels of immune-related molecules in TME, including chemokines and their receptors and immune checkpoints, were also significantly higher in IS1. In addition, IS1 showed a significantly higher immune score, and the two ISs showed different immune infiltration states. The composition of the tumor varies from patient to patient, and has a large degree of heterogeneity within itself (35). ScRNA-seq is a powerful strategy to study intratumor heterogeneity. When analyzing scRNA-seq data, we can cluster heterogeneous cell groups according to gene expression patterns and identify cell types by identifying specific gene markers of clustering (14). Here, through the analysis of 17,850 scRNA-seq data from 6 UM samples, we identified 11 cell clusters in UM, of which 2 were AXL+SIGLEC6+ dendritic cells, and the other 9 cell clusters represented different cells, including bipolar cells, macrophages, lymphocytes, amacrine cells, and different types of dendritic cells. By establishing the relationship between 11 cell clusters and ISs, we found that the marker genes of C0, C2, C3, C4, C6, and C10 were significantly enriched in IS2, indicating that IS2 may be rich in dendritic cells. The marker genes of C1, C7, C8, and C9 were significantly highly expressed in IS1. We screened five cell clusters (C1, C4, C5, C8, and C9) with significant differences in scores between IS1 and IS2 and were related to the prognosis of UM. According to trajectory analysis, they were projected onto three different differentiation states.

We also explored the transcriptional characteristics of five clusters. According to our analysis, JUN is a regulatory TF specifically expressed in CD1C-CD141- dendritic cells, and FOS is mainly expressed in CD1C-CD141- dendritic cells, macrophages, and mesenchymal stromal cells. SPI1 is the main regulatory TF of mesenchymal stromal cells. FOSB and JUNB are also important TFs in CD1C-CD141- dendritic cells. MAF is mainly expressed in macrophage and is a specific TF in macrophage. It is reported that both JUN and FOS are components of the activator protein-1 (AP-1) complex and play an important role in controlling the activity and function of dendritic cells (36, 37). The study published by Liu et al. showed that MAF was expressed in macrophages of human lung cancer and strictly regulates their immunosuppressive activity (38). These studies increase the credibility of our analysis results.

Finally, we also identified six key mRNAs (SLC2A3, CTNNB1, MAF, CYB5D2, RWDD1, and LDB2) through the construction of the mRNA–lncRNA interaction network. According to recent reports, SLC2A3 is a membrane transporter associated with EMT and immune characteristics in colorectal cancer (39), and its upregulation is associated with poor OS (40). The role and regulatory mechanism of CTNNB1 as a tumor marker in various cancers have also been reported by a number of studies (41–44). The TF MAF has been considered as a risk factor (HR for bone metastasis = 2.5, 95% CI = 1.7 to 3.8, *p* < 0.001) in metastatic breast cancer (45) and as a checkpoint modulating macrophage in lung cancer (38). The downregulation of CYB5D2 has been found to facilitate breast cancer progression (46). Limited studies were conducted to explore the role of the other two mRNAs (RWDD1 and LDB2) in cancer development. In addition, we also identified three key lncRNAs (EDRF1-DT, PSMA3-AS1, and TUG1) that closely interacted with the above mRNAs. PSMA3-AS1 and TUG1 have been reported to be involved in cancer development (47, 48).

Although we screened the six key mRNAs and three key lncRNAs that may be highly involved in UM development, only pure bioinformatics analysis was conducted in the present study. Further experiments in clinical UM samples should be implemented to verify our results. In addition, we did not consider other influence factors such as different stages that can affect intratumoral heterogeneity.

In summary, we analyzed the RNA-seq data and identified two immune subtypes of UM that showed different characteristics of OS and immune molecule and immune cell infiltration. Based on the analysis of scRNA-seq data, we identified 11 cell clusters, screened 5 cell clusters related to the prognosis of UM, and revealed their differentiation status and TF activity. Additionally, our study also selected hub genes through the construction of the mRNA–lncRNA interaction network, which not only emphasized the intertumor and intratumor heterogeneity of UM, but also provided a direction for the study of the UM regulatory mechanism.

## Data Availability Statement

The RNA-seq data of UM samples were obtained from TCGA (https://portal.gdc.cancer.gov/) database and GEO database (accession number: GSE22138, http://www.ncbi.nlm.nih.gov/geo/). The scRNA-seq data was also from the GEO database (accession number: GSE138433).

## Author Contributions

This study was designed by GG, SL, and SSL contributed to the literature research. XF and XZ analyzed and interpreted the data. AD wrote the initial draft of the manuscript. GG reviewed and edited the manuscript. All authors read and approved the manuscript.

## Funding

This study was supported by Shandong Medical and Health Science and Technology Development Program under grant number 2017WS245 and by China Disabled Persons’ Federation under grant number CJFJRRB19-2019.

## Conflict of Interest

The authors declare that the research was conducted in the absence of any commercial or financial relationships that could be construed as a potential conflict of interest.

## Publisher’s Note

All claims expressed in this article are solely those of the authors and do not necessarily represent those of their affiliated organizations, or those of the publisher, the editors and the reviewers. Any product that may be evaluated in this article, or claim that may be made by its manufacturer, is not guaranteed or endorsed by the publisher.
