# Supplementary figures and images for: Integration of Bulk RNA Sequencing and Single-Cell RNA Sequencing to Reveal Uveal Melanoma Tumor Heterogeneity and Cells Related to Survival
Source: Front Immunol. 2022 Jul 5;13:898925. doi: 10.3389/fimmu.2022.898925 (PMC9294459; doi:10.3389/fimmu.2022.898925)

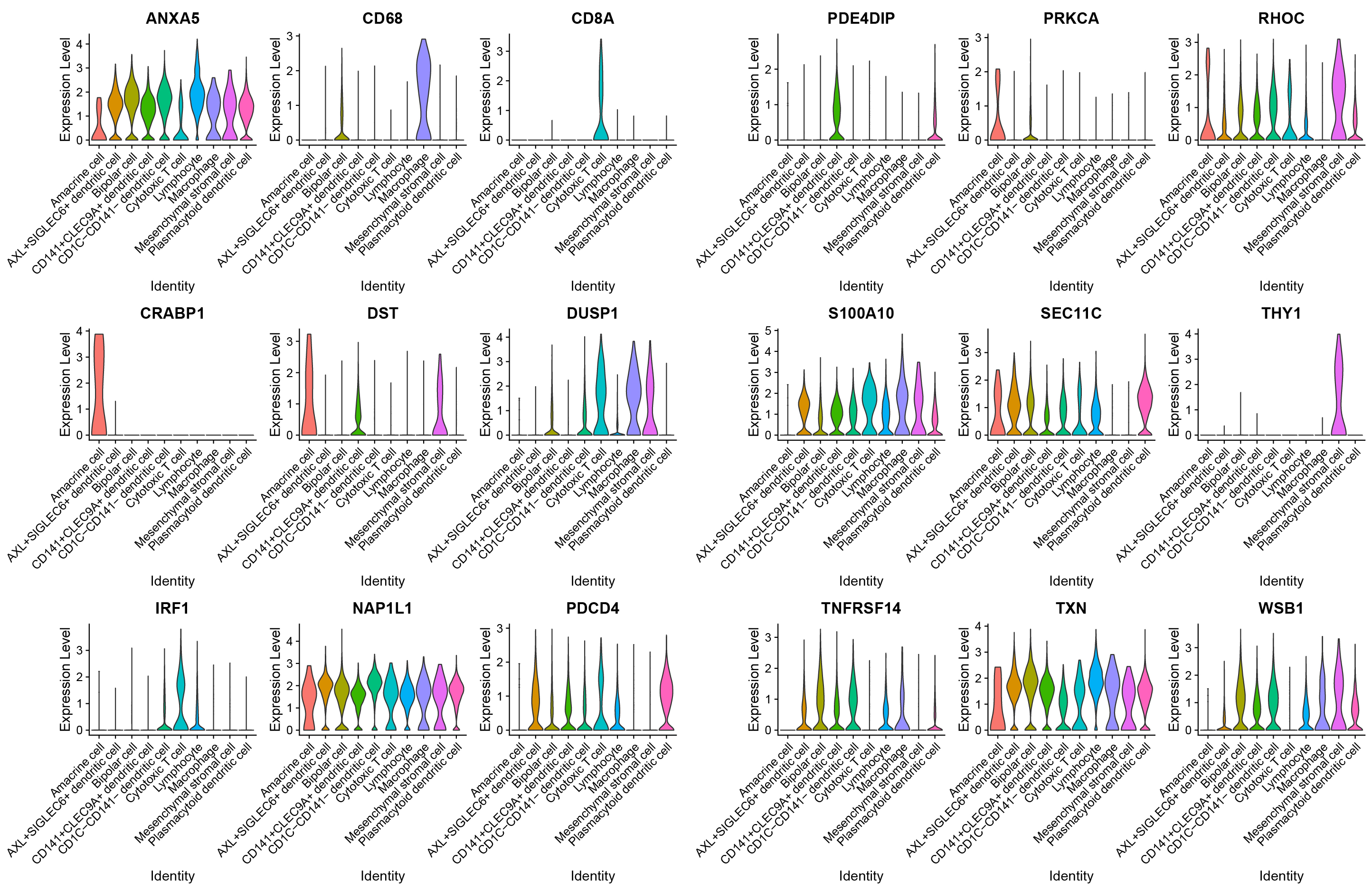

Supplement: Supplementary file 3 [file Image_1.jpeg]

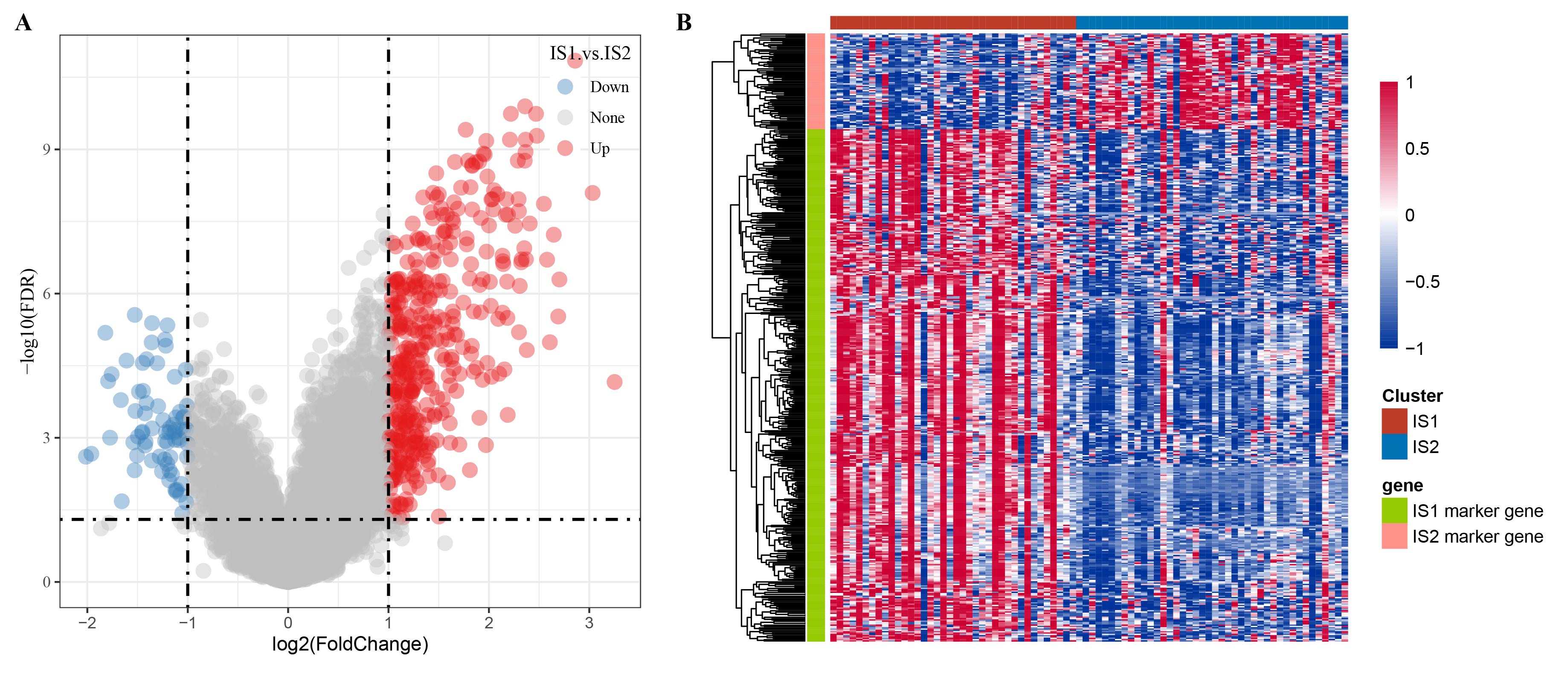

Supplement: Supplementary file 4 [file Image_2.jpeg]

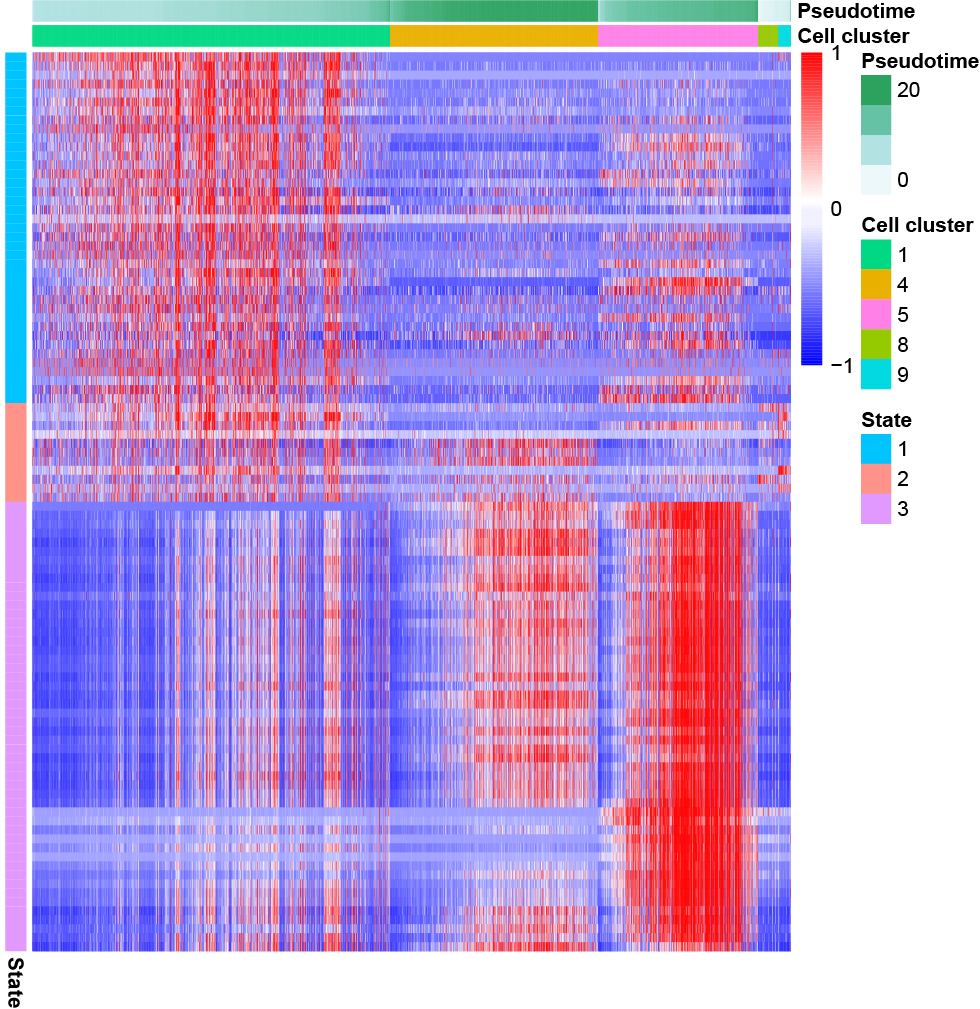

Supplement: Supplementary file 5 [file Image_3.jpeg]

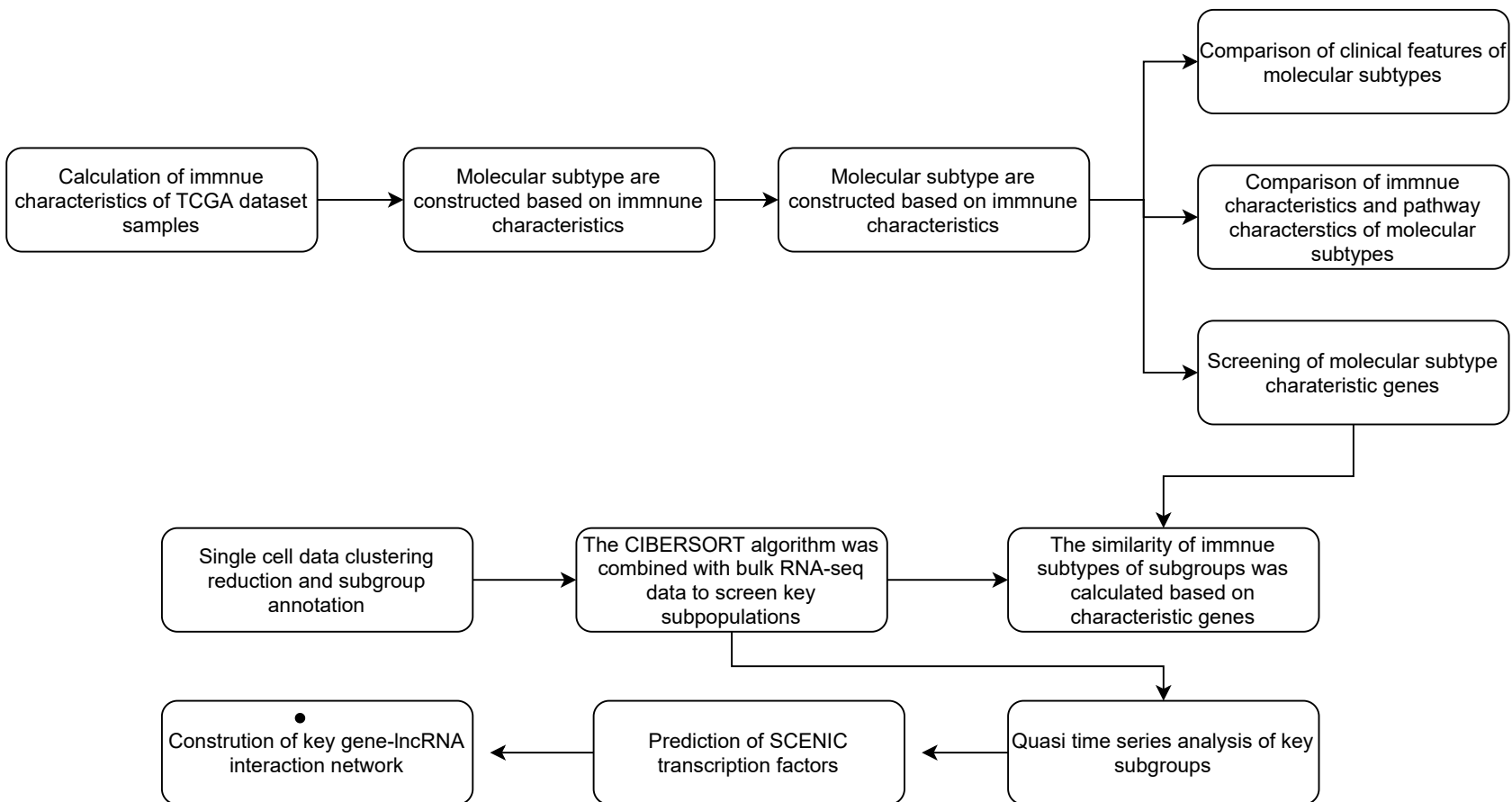

Supplement: Supplementary file 6 [file DataSheet_1.pdf]

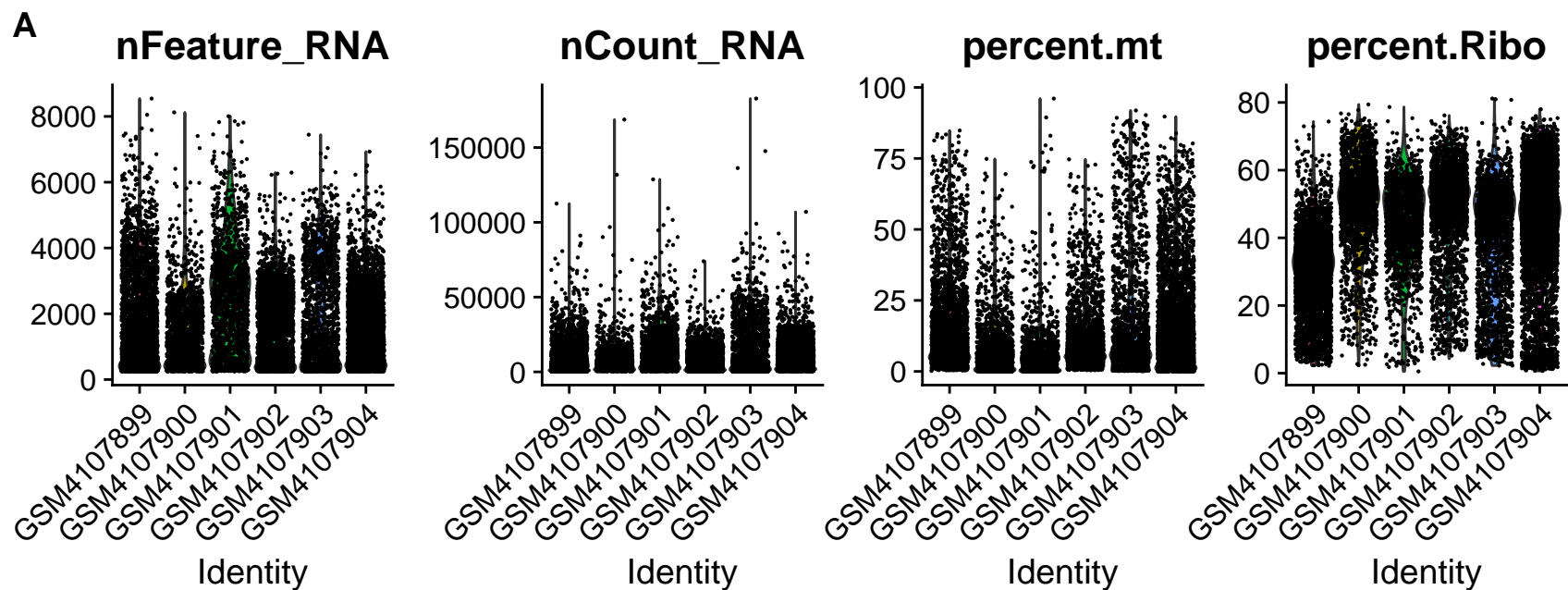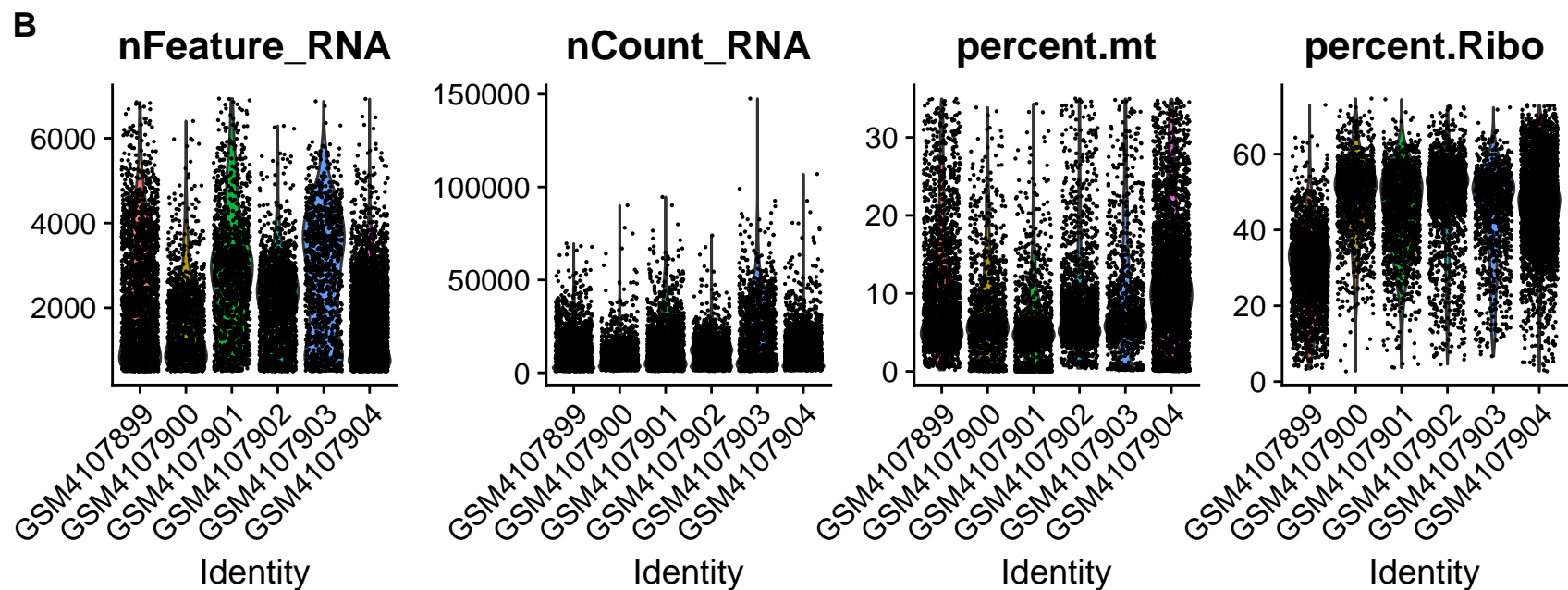

Supplement: Supplementary file 7 [file DataSheet_2.pdf]

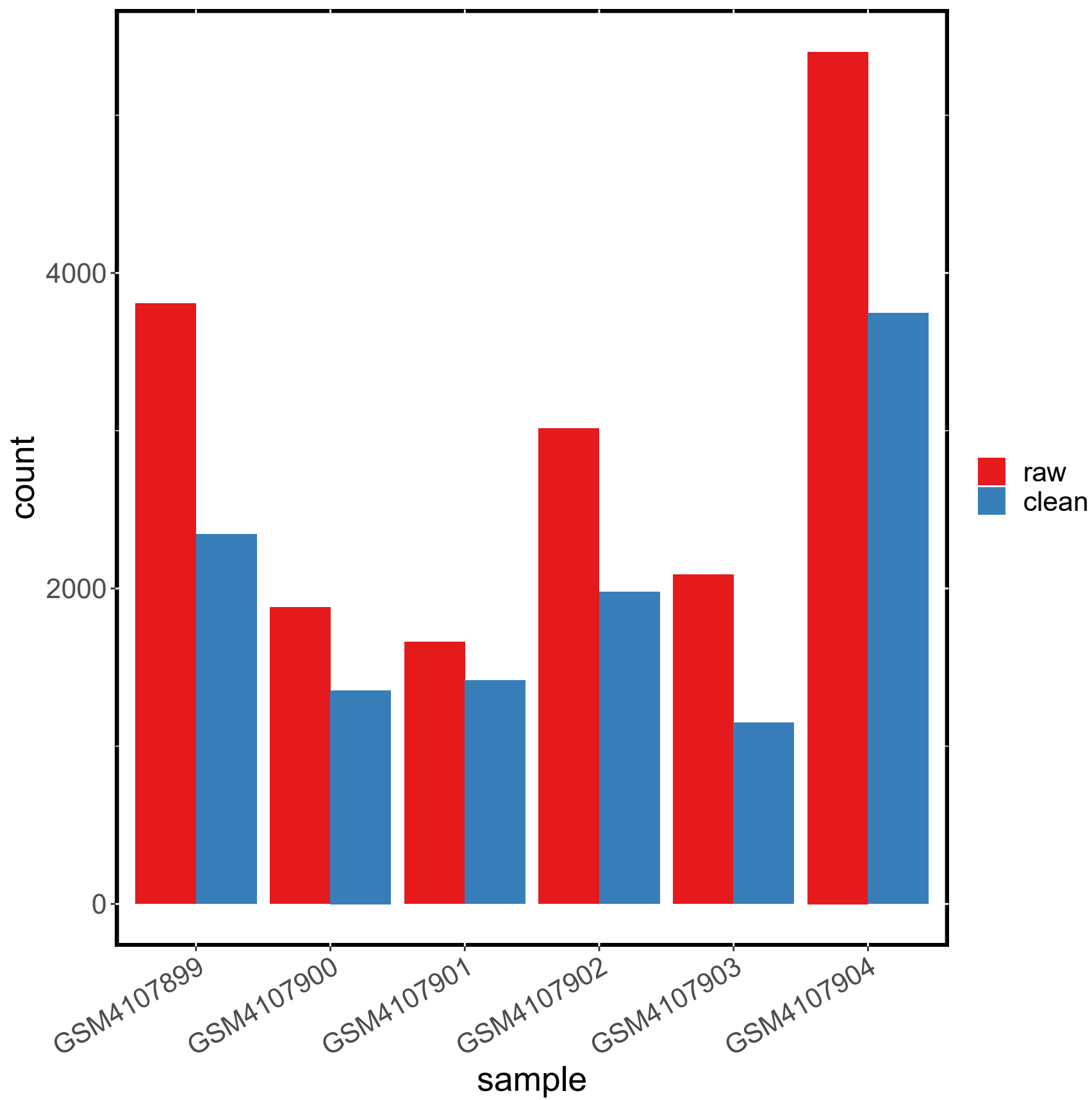

Supplement: Supplementary file 8 [file DataSheet_3.pdf]

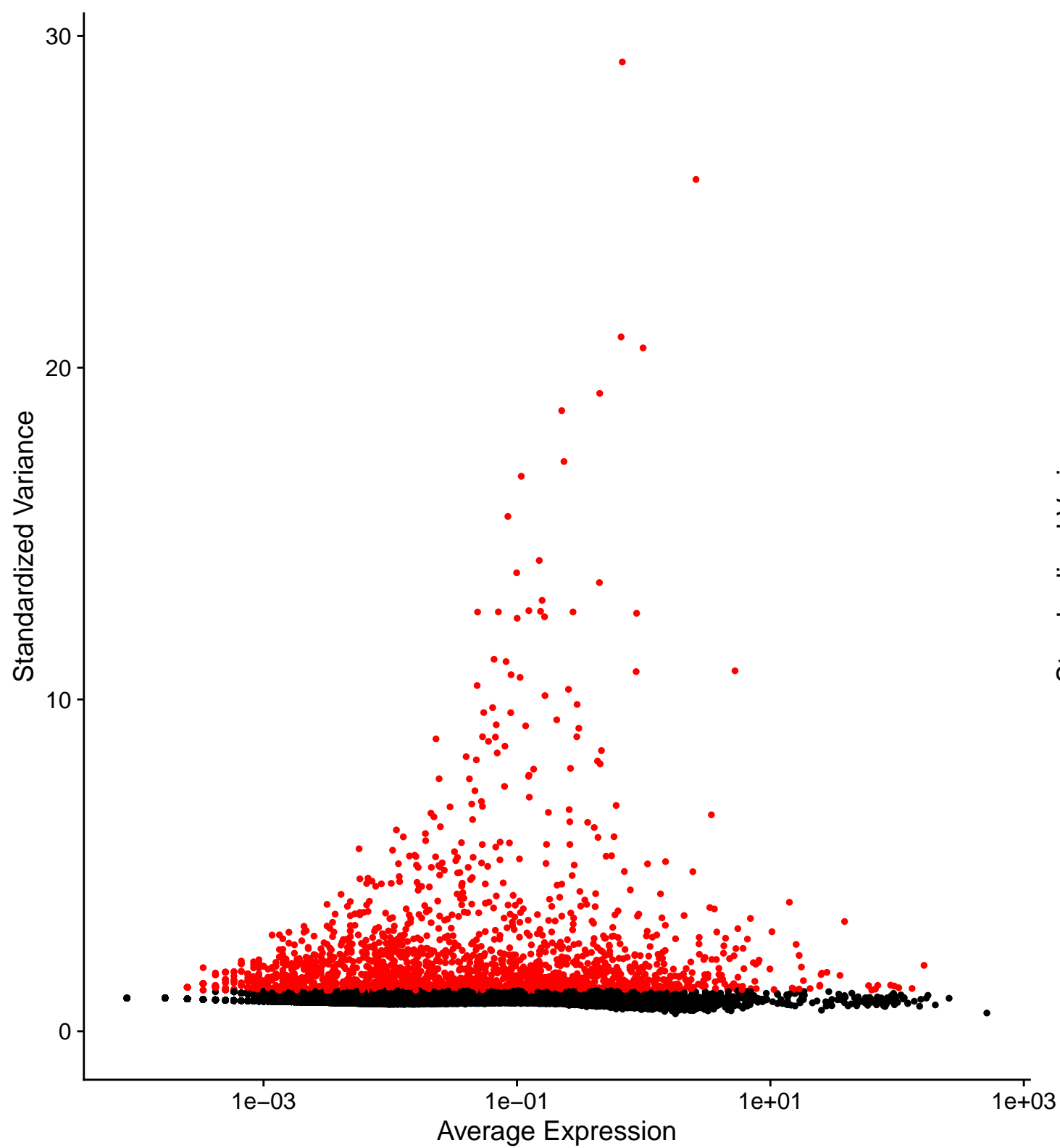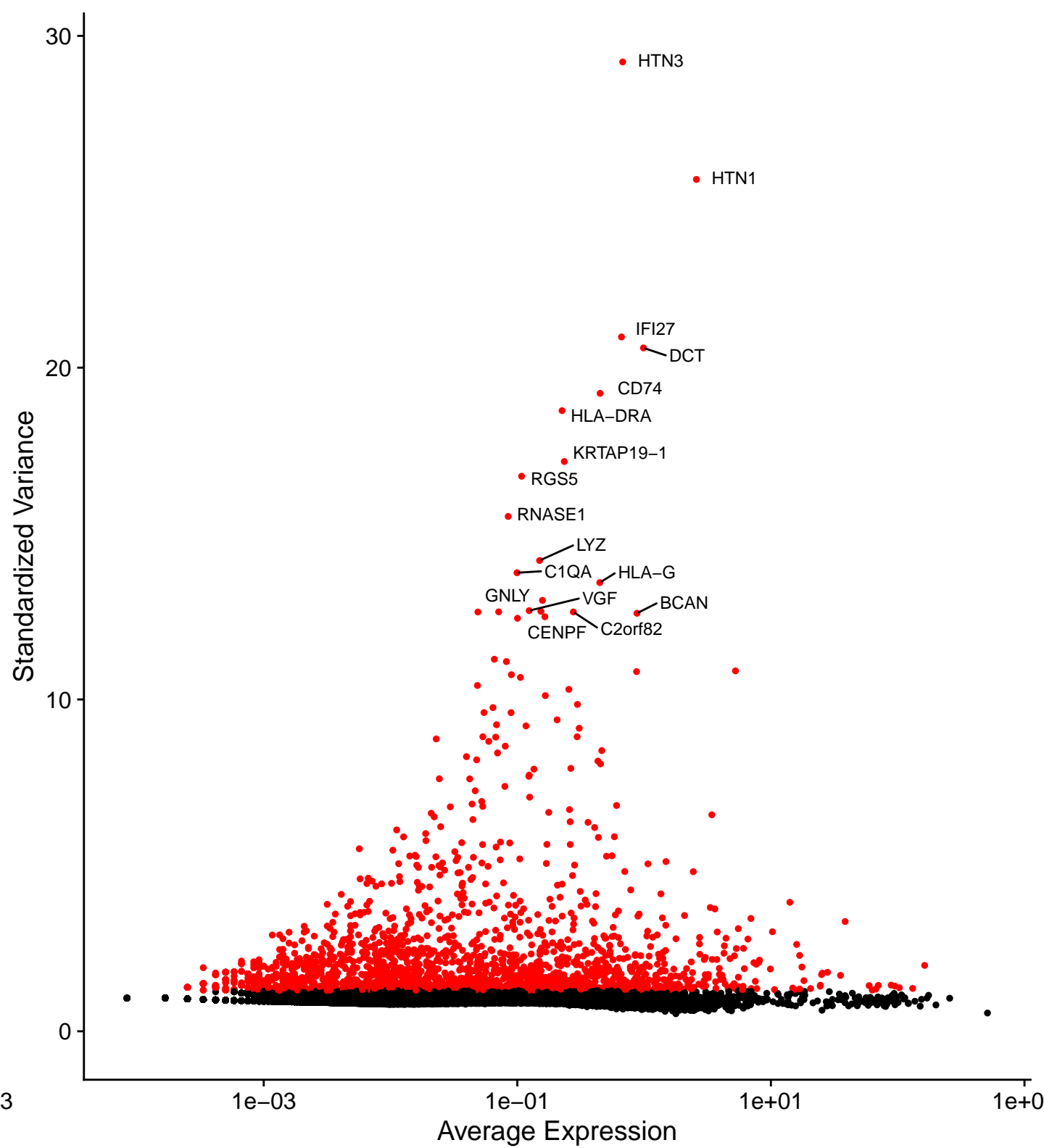

• Non-variable count: 18129 • Variable count: 2000

Supplement: Supplementary file 9 [file DataSheet_4.pdf]

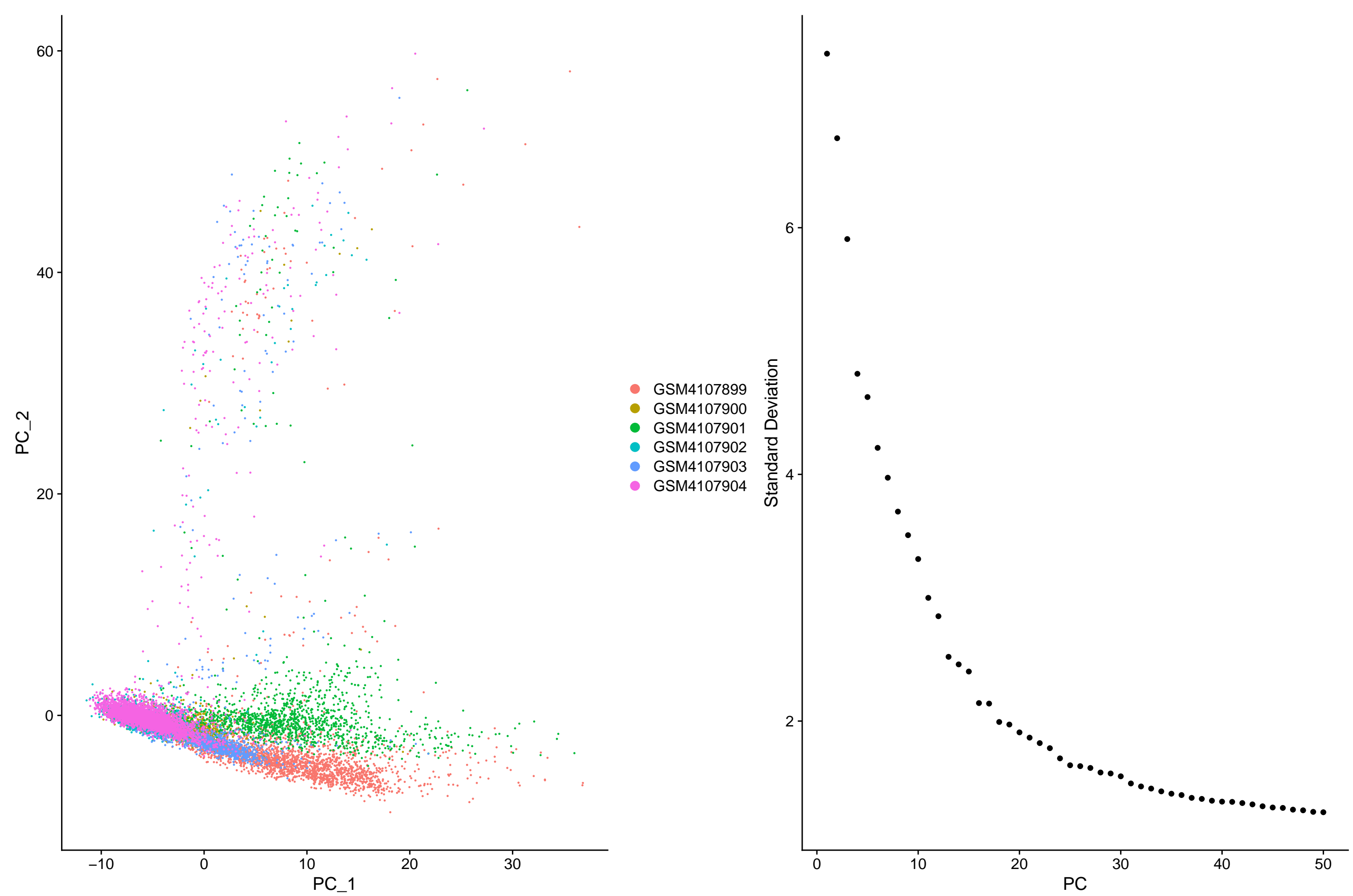

Supplement: Supplementary file 10 [file DataSheet_5.pdf]

IS1 vs IS2 differ gene

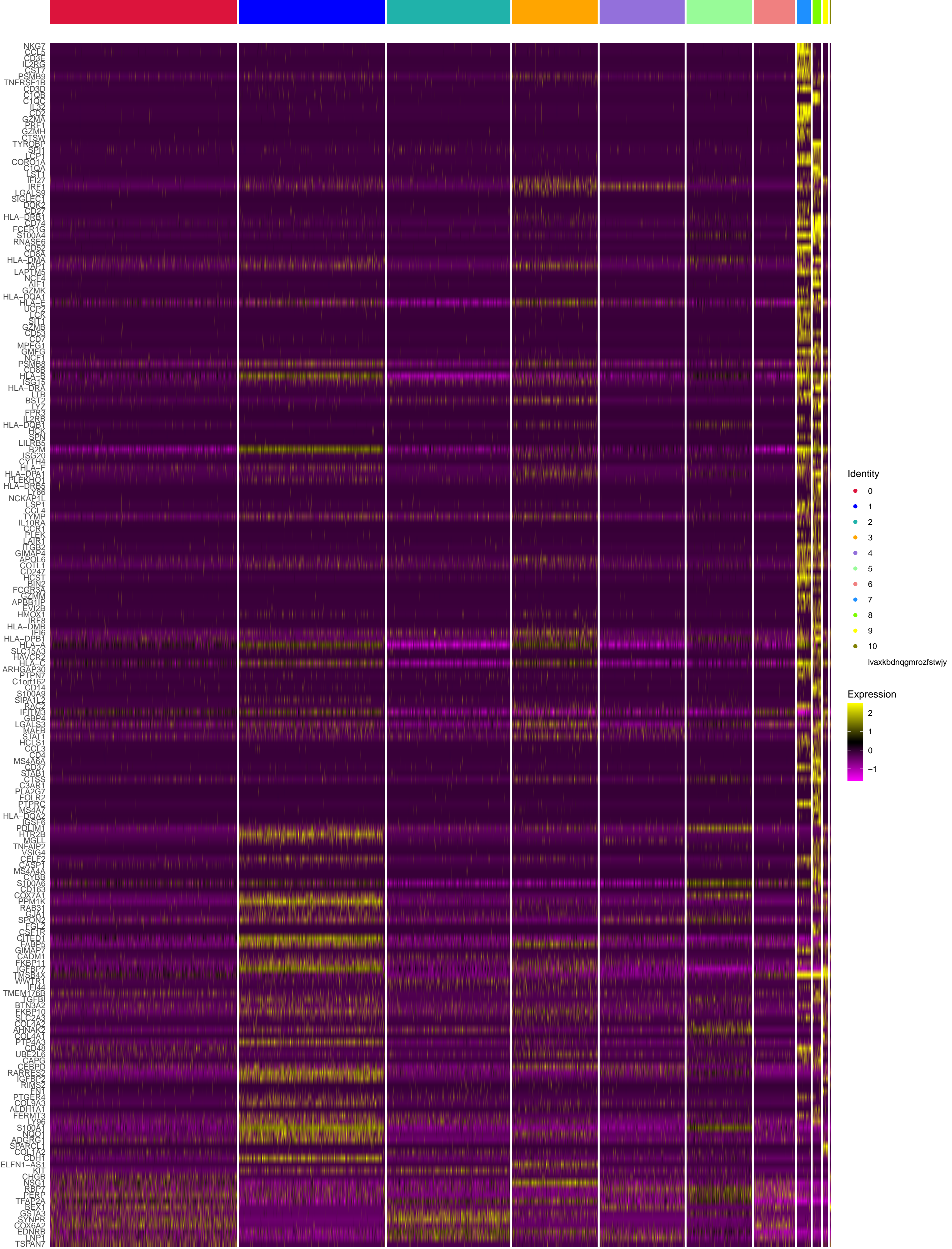

Supplement: Supplementary file 11 [file DataSheet_6.pdf]
